# Degenerative lumbar scoliosis with stenosis: how should Cobb angle inform decompression versus fusion? A systematic review

**DOI:** 10.1016/j.xnsj.2026.100919

**Published:** 2026-06-13

**Authors:** Deya AlWady, Spyridon Komaitis, Mohammed Shakil Patel, Khalid Salem, Nasir Quraishi, Konstantinos Zygogiannis

**Affiliations:** Centre for Spinal Studies and Surgery (CSSS) D Floor, West Block Queen's Medical Centre (QMC), Derby Road, Nottingham NG7 2UH, United Kingdom

**Keywords:** Degenerative lumbar scoliosis, Lumbar stenosis, Cobb angle, Decompression, Fusion, Adult spinal deformity

## Abstract

**Background:**

Degenerative lumbar scoliosis (DLS) with stenosis is a three-dimensional disorder in which the magnitude of the coronal curve informs, but does not, by itself, determine operative planning.

**Objective:**

To assess how the preoperative Cobb angle should be interpreted when choosing decompression alone versus fusion in adults with DLS and stenosis.

**Methods:**

Ovid MEDLINE and Embase databases were searched from 1996 to April 25, 2025. The search in Ovid was created by combining keyword and subject heading terms for adult degenerative scoliosis/adult spinal deformity, lumbar scoliosis, Cobb angle, stenosis, decompression, and fusion. Studies eligible for inclusion were adult surgical cohorts that either provided explicit data on preoperative Cobb angles or included patients based on a preoperative Cobb angle threshold, and that reported at least one outcome measure following surgery for decompression and/or fusion. Cohorts consisting of mixed populations were excluded unless an analysis of the appropriate subset of adults with DLS could be performed independently. Heterogeneous studies were synthesized using narrative methods.

**Results:**

Four observational studies met the inclusion criteria. In mild curves, generally below 20°, decompression alone produced acceptable outcomes when stenosis predominated and instability was limited. In the limited comparative evidence, fusion appeared more favorable when curves were larger or when mechanical features such as instability or more substantial deformity were present. Across the included studies, important modifiers beyond Cobb angle included foraminal stenosis, lateral listhesis, disc wedging, and sagittal alignment. Follow-up ranged from approximately 1 to 4.6 years.

**Conclusions:**

Cobb angle is a useful decision aid, but not a stand-alone indication. Operative choice in DLS with stenosis should integrate curve magnitude with stenosis pattern, instability, sagittal alignment, symptoms, comorbidity, frailty, and the expected burden of surgery.

## Introduction

Degenerative lumbar scoliosis (DLS), an adult spinal deformity, typically occurs after skeletal maturity due to asymmetric degeneration of the facet joints and intervertebral discs. While the coronal Cobb angle is the most widely used measure of curvature in DLS, it represents only 1 aspect of this condition; axial rotation, lateral translation, segmental listhesis, and sagittal-plane misalignment can also contribute to both symptoms and surgical planning. In fact, while much of the published literature on adult scoliosis includes cases of DLS and adult idiopathic scoliosis together, this study addresses DLS alone with stenotic symptoms [1,2].

Similarly, the symptomatic expression of DLS with stenosis involves multiple factors. Pain due to radiculopathy and neurogenic claudication are common manifestations, but fixed neurological deficits are less frequently encountered. Neural compression may arise from several mechanisms, including asymmetrically collapsed intervertebral discs, hypertrophied facets, thickened ligamentum flavum, rotary subluxations, and narrowed foramina at mechanically stressed levels. Ferrero et al. have shown a predictable relationship between stenosis location and curve morphology. For example, central stenosis tends to occur near the transition zone of the lumbar and lumbosacral curves, whereas foraminal and lateral stenosis tend to occur in the concave side of the distal lumbosacral curve. This provides insight into why symptom locations and global coronal curvature magnitudes do not correlate perfectly [1,3].

Due to the combination of deformity and localized stenosis seen in DLS, operative treatment options vary widely. When symptoms are caused primarily by focal neural compression at a small, well-balanced, and relatively stable curve and when the patient is elderly or has other medical conditions that make major surgery risky, limited decompressive procedures may provide sufficient relief. Conversely, when focal neural compression coexists with lateral listhesis, disc wedging, rotatory subluxation, or local sagittal plane asymmetry at the symptomatic level, short-fusion is usually preferred. Finally, long-segment deformity corrective fusions remain applicable for selected patients who suffer significant coronal or sagittal imbalance or progressive deformity or those whose primary complaints include significant mechanical low back pain. However, these latter patients must be carefully evaluated to determine whether their anticipated benefits outweigh the increased physiological burdens associated with advancing age, decreased physical resilience, the presence of comorbidities, or specific treatment objectives. Therefore, full-length standing radiographs are necessary to place localized stenosis in context relative to overall deformity patterns [2,[4], [5], [6], [7], [8], [9], [10], [11]].

Recent publications recognize that no single radiographic measurement is likely to be universally applicable in determining which DLS patients will benefit from surgical intervention. Broader systematic reviews increasingly support evidence-based contextual decisions regarding individual treatments; however, they do not address how the preoperative coronal Cobb angle should be interpreted in studies reporting preoperative coronal magnitudes in DLS patients presenting with stenosis [12]. Therefore, our aim was to investigate the following research question: What preoperative Cobb angle range in adult patients with DLS and stenosis supports limited decompressive surgery alone, and which clinical/radiographic variables modify treatment to either limited or extensive (fusional) surgical intervention? Our hypothesis was that, based on the existing literature, no universal Cobb angle cutoff would be identified to guide surgeons' decisions regarding instability, stenosis patterns, sagittal alignment, and patients' frailty status.

## Materials and methods

This systematic review followed the Preferred Reporting Items for Systematic Reviews and Meta-Analyses (PRISMA) 2020 guidelines [13]. We searched Ovid MEDLINE and Embase for studies published from 1996 to April 2025 and also examined the reference lists for potentially relevant studies.

Ovid's search strategy included indexed subject headings and free-text terms for both the disease and the decision factor, as well as for symptomatology and treatments. Practically speaking, the searches used combinations of adult degenerative scoliosis OR adult spinal deformity OR lumbar scoliosis with Cobb angle OR coronal angle, in combination with stenosis OR lumbar stenosis, and then again in combination with decompression OR fusion OR arthrodesis OR minimally invasive surgery. This strategy was designed to be broader in scope than “adult degenerative lumbar scoliosis” alone to ensure that potentially relevant DLS cohorts under other deformity terminology would not be overlooked.

For this review, DLS was defined as adult degenerative lumbar or thoracolumbar scoliosis with a minimum standing coronal Cobb angle of 10 degrees. Studies that focused solely on longstanding idiopathic scoliosis were excluded unless they provided an opportunity to analyze a separate subgroup of DLS patients. Eligible cohorts had to have reported a preoperative Cobb angle explicitly or use a preoperative Cobb angle threshold as part of their selection process in order to align with the review question. Although studies providing insight into the pathology or distribution of stenosis were referenced as background literature, they did not provide sufficient postoperative surgical outcome data to be considered in our qualitative analysis of surgical interventions [1,3].

Inclusion criteria were: adult surgical cohorts that included DLS or stenosis-associated degenerative lumbar coronal deformities relevant to DLS decision-making; explicit reporting of preoperative Cobb angles or an expressed preoperative Cobb angle threshold; and at least 1 postoperative surgical-related outcome concerning operative choice, which includes: functional outcome; radiographic progression; reoperation; or mechanical complications. Exclusion criteria were: medical interventional studies; pediatric or adolescent scoliosis; mixed adult scoliosis cohorts without a separable DLS subgroup; studies without available preoperative Cobb angle data; studies without postoperative surgical outcome data; and technical notes, case reports, and editorials.

Two independent reviewers evaluated titles and abstracts for inclusion, reviewed the full-text articles for compliance with the eligibility criteria, and collected data on study design, number of patients in each study group, age, Cobb angle context, surgical approach, duration of follow-up, and the primary conclusion drawn by the authors for each study. Any disagreements among the 2 reviewers were resolved through discussion until a consensus was reached. As all eligible studies were observational and reported heterogeneous indications and outcomes, we selected a narrative synthesis based on the domains of reported outcomes rather than conducting a formal meta-analytic evaluation. Each study was qualitatively assessed for robustness based on the specificity of the cohort, the availability of comparative groups, the length of follow-up, and the completeness of clinical and radiographic outcome reporting.

**Fig. 1:**
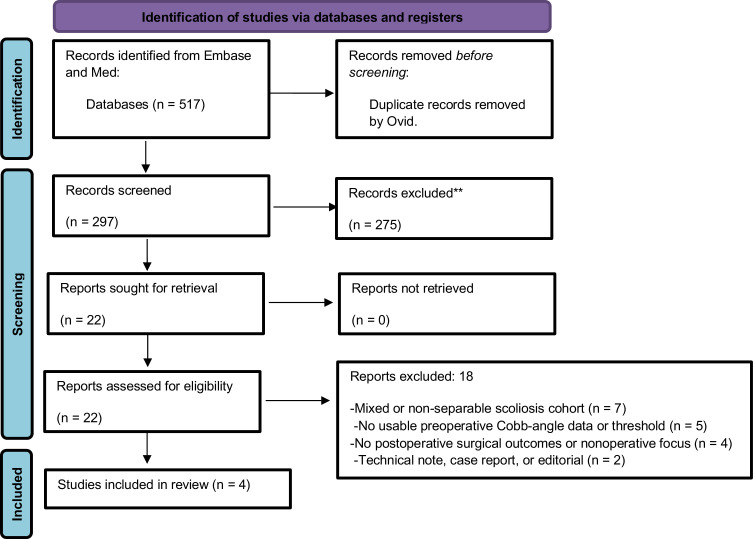
PRISMA 2020 flow diagram for study selection.

The complete study-selection pathway is summarized in Figure 1.

## Results

### Study selection and overall characteristics

A total of 517 records were retrieved in the literature review. Of those, 220 duplicate records were removed. A total of 297 titles/abstracts were reviewed, and 275 of those records were excluded. Twenty-two full-text articles were reviewed. Of those 22 articles reviewed, 18 were excluded. Therefore, a total of 4 full-text articles were included in the final qualitative analysis. The 4 studies that met the inclusion criteria were all observational cohorts. Two of the studies were comparative cohort studies, and 2 were decompression-only cohort studies. There were no randomized studies. Follow-up time varied across the included studies. Time frames ranged from approximately 1 to 4.6 years [[4], [5], [6], [7]].

### Study-level findings

Daubs et al. provided the longest follow-up of any comparative study. This study investigated elderly patients with mild DLS who underwent either decompression only or decompression with limited fusion. The mean preoperative Cobb angle of the decompression group was less than the mean preoperative Cobb angle of the fusion group. Also, this study indicated that limited fusion could potentially reduce recurrent stenotic symptoms in certain patient populations with more mechanically relevant deformities [4]. Kato et al. [5] also investigated a minimally invasive decompression cohort of patients. In this cohort, the DLS subgroup had an average Cobb angle of 13.8°. Lateral listhesis and disc wedging emerged as significant predictors of reoperation. The fact that instability modified the relative importance of what would otherwise appear to be a relatively small coronal curve further supports these conclusions [5]. Likewise, Minamide et al. [6] investigated minimally invasive decompression in patients with DLS. They concluded that outcomes are worse when severe scoliosis, foraminal stenosis, progressive scoliosis, or a large pelvic incidence-lumbar lordosis mismatch is present. Asada et al. [7] investigated patients with lumbar canal stenosis and moderate degenerative coronal deformity (Cobb angle >20°) and found that short-segment fusion provided greater short-term improvement in disability and back pain than isolated decompression.

### Clinical outcomes and symptom relief

All 4 studies included in the current systematic review demonstrated clinically significant improvements in symptoms when surgery was performed to address stenosis as the primary complaint. Decompression-only series in carefully selected mild DLS cases demonstrated clinically significant improvements in pain and functional status, while comparative cohorts showed that both decompression-focused and fusion-based surgical options can be effective when they match the mechanical profile of the deformity. The common theme across studies was that neither Cobb angle nor other radiographic measures independently determined success. Rather, decompression was most successful in smaller, relatively stable curves, while fusion became more desirable as deformity or instability increased [[4], [5], [6], [7]].

### Perioperative burden and morbidity

As stated previously, the minimally invasive decompression series demonstrates the lower perioperative burden associated with decompression-focused surgeries. Conversely, the comparative cohorts suggest that fusion provides better structural support or radiographic control for higher operative burdens. Ultimately, age, comorbid conditions, and frailty continue to be the guiding principles for determining whether to pursue a decompression-focused or fusion-based operation, especially when the radiographic situation falls within a gray area [4,6,7,11].

### Radiographic instability and modifiers beyond Cobb angle

The included studies consistently demonstrated that Cobb magnitude alone was inadequate for operative planning purposes. Specifically, poorer outcomes or higher reoperation risks were observed in cases with foraminal stenosis, progressive scoliosis, large pelvic incidence-lumbar lordosis mismatch, lateral listhesis, or disc wedging. These modifiers help explain why patients with identical coronal curves may require different operative approaches and why a strictly threshold-based approach is overly simplistic [[5], [6], [7], [8], [9]].

### Follow-up and durability

Importantly, the range of follow-up times across the included studies (approximately 1–4.6 years) has implications for evaluating durability. While short-term stability after decompression is encouraging, it does not preclude potential future progression or recurrent symptoms. Longer follow-up in Daubs et al. provided greater insight into recurrence than comparative data collected over shorter follow-up periods, while the decompression-only cohorts provided more information about the natural history of mildly selected curves [[4], [5], [6], [7]].

Design, Cobb-angle context, duration of follow-up, and primary decision-making message for each included study are summarized in Table 1 below.

**Table 1:** Included studies and principal decision-making messages.

Study	Design/cohort	Cobb-angle context	Surgical comparison	Follow-up	Principal message
Daubs et al. [4]	Retrospective comparative; elderly mild DLS	Mean Cobb 16° in decompression group; 22° in limited-fusion group	Decompression alone vs decompression with limited fusion	4.6 years	Limited fusion reduced recurrent stenotic symptoms in selected patients; decompression alone remained most defensible in mild, stable curves.
Kato et al. [5]	Prospective minimally invasive decompression cohort; DLS subgroup analyzed	Mean Cobb 13.8° (range 10°–18°)	Decompression alone	About 2 years	Lateral listhesis and disc wedging predicted reoperation, showing that instability modifies the meaning of a small coronal curve.
Minamide et al. [6]	Prospective minimally invasive decompression cohort	Mean Cobb 16.3° ± 7.1°	Decompression alone	2.4 years	Poorer outcomes were linked to severe scoliosis, foraminal stenosis, progressive scoliosis, and PI-LL mismatch.
Asada et al. [7]	Retrospective comparative; lumbar canal stenosis with moderate degenerative coronal deformity	>20° inclusion threshold	Isolated decompression vs short-segment fusion	1 year	In patients with Cobb angles >20°, short-segment fusion produced better short-term disability and back pain improvement than isolated decompression.

DLS, degenerative lumbar scoliosis; PI-LL, pelvic incidence-lumbar lordosis.

## Discussion

### Meaningful interpretation

While there is strong evidence to support a “measured” definition of the Cobb angle for operative decision-making, the Cobb angle still has value as an initial step in decision-making. However, while the Cobb angle may remain a useful parameter in evaluating candidates for operative treatment, it should not be considered the sole factor in determining whether to proceed with surgery. Rather, a surgeon would need to evaluate the Cobb angle in the context of the stenosis pattern, instability, sagittal alignment, symptoms, age, comorbidities, and frailty. While each of the 4 cohorts included in the analysis identified no universally applicable biological or surgical thresholds, they did demonstrate that decompression alone is indicated for small, mechanically silent curves, while fusion is indicated when deformity or instability reaches clinically relevant levels [[4], [5], [6], [7]].

### Comparisons with broader literature

The evidence provided herein regarding the lack of a single stand-alone coronal threshold for operative indications is consistent with the broad adult scoliosis and systematic review literatures that also argue against a single stand-alone coronal threshold. The systematic reviews by Echt et al. [12], however, evaluated a more heterogeneous population of adults with deformities (some studies included a wide variety of interventions) and are thus unable to address the narrow question at hand. The current review provides additional information, as it uses a more conservative inclusion criterion based on the Cobb angle, but that same conservatism limits the number of studies that meet those criteria.

### DLS with stenosis is not only a coronal problem

These findings must be interpreted in the context of DLS as a 3-dimensional stenotic deformity, not just a simple coronal curve. Ferrero et al. [3] demonstrated that stenosis locations depend on the curve morphology. This illustrates why a patient with a relatively modest Cobb angle may experience significant radicular symptoms if their symptomatic level resides in a mechanically disadvantageous portion of their curve [3]. For this reason, the Cobb angle cannot independently predict either symptom severity or the necessity for operative intervention.

### Long fusion and instability, sagittal alignment

Signals in Kato, Minamide, and Asada are consistent with both older radiographic and surgical literature, demonstrating that lateral listhesis, disc wedging, rotatory subluxation, and sagittal imbalance significantly affect outcomes [[5], [6], [7], [8], [9]]. Additionally, broader deformity literature demonstrates that long fusion will benefit patients who have greater curvature or a more global imbalance of their deformities. However, the literature highlights the increased physiological burden associated with longer fusions [10,11,14]. Accordingly, decisions to increase the length of fusion from decompression alone to short fusion and ultimately to long fusion should consider not only coronal magnitudes, but also mechanical behavior, global alignment, and physiologic reserves.

### Follow-up horizon and frailty

Both concerns are valid. Restoring alignment is not simply a radiographic outcome; it must also be balanced with age, comorbidities, and frailty [11]. Similarly, limited curve progression at 1 or 2 years should not be misinterpreted as sufficient evidence that subsequent deformity progression is clinically insignificant. Both Hwang et al. and the comparative cohorts illustrate how postoperative curve behavior may evolve over time––particularly when the mechanical aspects of deformity are inadequately corrected [4,15].

### Limitations of review

As mentioned previously, this review has many limitations due to the quality and quantity of available research. Specifically, all reviewed articles were observational. Most of them originated from a single center. Indications for operation varied among articles. Although important variables such as frailty and global sagittal balance were frequently omitted, there was variability in the uniformity of their documentation across studies. One comparative study selected for review involved a mixed population of patients with lumbar spinal stenosis and moderate degenerative coronal deformity defined by Cobb angle versus a classically labeled de novo DLS cohort. We chose to retain it because it directly compared decompression with fusion in the context of stenosis and coronal deformity examined here. However, it should be viewed through this lens. Our decision to apply a strict inclusion criterion requiring explicit documentation of preoperative Cobb angle values or cohort definitions based on thresholds resulted in the exclusion of multiple other potentially relevant adult deformity articles. We made this trade-off to keep our review focused on the specific clinical question under examination. Each limitation warrants presentation in a nonquantitative format and precludes a claim of evidence-based universal application of a single Cobb angle threshold [[4], [5], [6], [7],^12^].

Table 2 presents the findings above in a practical multiparameter approach to assessing candidates for operative treatment using Cobb angle, along with stenosis patterns, instability, alignment, and the candidate’s frailty, rather than an individual cutoff.

**Table 2:** Practical multiparameter interpretation of Cobb angle in DLS with stenosis.

Cobb magnitude	Stenosis/stability pattern	Patient factors	Usually favored strategy	Interpretive caution
<20°	Focal canal/lateral recess stenosis; no major listhesis or rotatory subluxation; acceptable alignment	Older or frail patient; predominant radicular pain or claudication; limited mechanical back pain	Usually decompression alone	Best supported scenario in the retained evidence for symptom-directed decompression.
20°–30°	Assess foraminal stenosis, disc wedging, lateral listhesis, PI-LL mismatch, and symptomatic level	Shared decision-making; comorbidity and frailty influence how much surgery is reasonable	Individualized: decompression alone or short fusion	Gray zone in which mechanical modifiers matter more than a single coronal threshold.
Clear instability or broader imbalance (regardless of a single-angle cutoff)	More likely mechanical deformity, coronal/sagittal imbalance, or recurrent instability	Fitter patient may benefit from more extensive correction; frailer patient may still need palliative surgery	Fusion is generally favored; long fusion is considered selectively	This inference is better supported by the broader comparative literature than by the 4 retained Cobb-specific studies alone.

DLS, degenerative lumbar scoliosis; PI-LL, pelvic incidence-lumbar lordosis.

## Conclusions

Preoperative measurement of the coronal Cobb angle can be used as one of many factors to assist in decision-making about surgery; it cannot be relied on solely as an indicator of the need for spinal decompression or stabilization. Decompression (alone) may be the best option to address a symptomatic degree of lumbar stenosis in adults if the curve measured by Cobb’s method is relatively low, if the central stenosis is localized to a specific area, if sagittal balance is preserved and there are no significant listhesis, subluxations or rotational deformities at the time of examination and/or during testing and there is little to no predominant mechanical back pain. There is some, albeit limited, comparative research suggesting that curves >20°, combined with other mechanical issues associated with lumbar spinal stenosis, favor surgical intervention; however, none of these studies has provided a universally accepted cut-off value for this assessment. Therefore, decisions regarding operative interventions for individuals diagnosed with DLS should continue to be made based upon individualized criteria, including symptomatology, stability, frailty status, and desired outcomes.

## Authorship Contribution Statement

**Deya AlWady:** Conceptualization, Methodology, Investigation, Data curation, Formal analysis, Writing—original draft, Visualization. **Konstantinos Zygogiannis:** Conceptualization, Methodology, Supervision, Writing—review and editing, Project administration. **Spyridon Komaitis:** Data curation, Investigation, Writing—review and editing. **Mohammed Shakil Patel:** Formal analysis, Validation, Writing—review and editing. **Khalid Salem:** Investigation, Resources, Writing—review and editing. **Nasir Quraishi:** Supervision, Validation, Writing—review and editing.

## Declarations of competing interests

The authors declare that they have no known competing financial interests or personal relationships that could have appeared to influence the work reported in this paper.
